# Contamination Status, Environmental Factor and Risk Assessment of Polychlorinated Biphenyls and Hexachlorobutadiene in Greenhouse and Open-Field Agricultural Soils across China

**DOI:** 10.3390/toxics11110941

**Published:** 2023-11-20

**Authors:** Yaru Li, Fangwei Hou, Rongguang Shi, Xiaohua Li, Jing Lan, Zongshan Zhao

**Affiliations:** 1 College of Environmental Science and Engineering, Qingdao University, Qingdao 266071, China; yrli@qdu.edu.cn (Y.L.); zhaozs@qdu.edu.cn (Z.Z.); 2 College of Mechanical and Electrical Engineering, Qingdao University, Qingdao 266071, China; houfw@qdu.edu.cn; 3 Agro-Environmental Protection Institute, Ministry of Agriculture and Rural Affairs, Tianjin 300191, China; winsomesky@163.com; 4 Rural Energy & Environment Agency, Ministry of Agriculture and Rural Affairs, Beijing 100125, China; lixiaohua8008@126.com

**Keywords:** polychlorinated biphenyls, hexachlorobutadiene, agricultural soils, contamination status, risk assessments

## Abstract

With the popularization and high-intensity utilization of greenhouse cultivation for crops growth, the pollution of greenhouse soils has been of concern. Therefore, a national-scale survey was conducted to investigate the contamination status, sources, influence factors and the risks of polychlorinated biphenyls (PCBs) and hexachlorobutadiene (HCBD) in greenhouse and nearby open-field soils. Contents of PCBs ranged from <LOD to 673.78 ng/g (mean: 77.38 ng/g) in greenhouse soils, and <LOD to 552.53 ng/g (mean: 61.90 ng/g) in open-field soils. HCBD was detected in all greenhouse soils with content ranging from 0.85 to 24.18 ng/g (mean: 8.33 ng/g), and a range of <LOD-20.19 ng/g (mean: 6.39 ng/g) in open-field soils. The sources of PCBs were the disposal of electrical equipment, domestic coal, wood burning emissions, etc. Levels of PCBs and HCBD were not correlated with the soil properties but positively correlated with *Pseudomonas* as the PCBs-degrader in open-field soils. Although the higher values of mean contents were found in greenhouses, the health risks of ΣPCBs in open-field soils were higher than in greenhouse soils due to the higher percentages of high-toxicity PCBs, especially the carcinogenic risks to children (>10^−6^). This study provided a full insight on the contamination status and risks of PCBs and HCBD when guiding greenhouse agriculture activities.

## 1. Introduction

Two cultivation methods (greenhouse and open-field) are generally used for crop production in agriculture. Greenhouse, characterized by a strong risk resistance, highly concentrated knowledge, and technology, has brought multiple benefits to the economy, society and ecosystems [1,2]. It has been widely employed to enhance the production of vegetables, fruits and flowers as stated by the Food and Agriculture Organization of the United Nations [3,4]. Since its first introduction in China during the late 1970s, the total area of greenhouse cultivation projects reached 4.708 million hectares in 2019 [5]. The improved yields and the shortened production cycle are usually accompanied with the usage of plenty of fertilizers, pesticides, energy and other materials, resulting in more serious soil contamination in greenhouse projects [6]. For example, higher levels of some persistent organic pollutants (POPs) were found in greenhouse soils than in open-field soils, such as pyrethroids, atrazine, organophosphorus pesticides, phthalate esters and polycyclic aromatic hydrocarbon (PAHs) [7,8,9]. Thus, it is necessary to determine the contamination status and risk of pollutants in greenhouse and open-field soils within a national-scale to comprehensively evaluate the agroecological effect.

Polychlorinated biphenyls (PCBs) and hexachlorobutadiene (HCBD), both belonging to POPs, are toxic legacy chemicals that could resist degradation in the environment and bioaccumulate in organisms [10,11]. They have been widely utilized in various applications, such as heat-exchange fluids, dielectric materials, pesticide additives, transformers, herbicides, and fungicides [12,13,14,15]. With improper disposal, unintentional leakage, and environmental migration, they can enter soils through irrigation wastewaters, chemical fertilizer, atmospheric deposition, etc., and have been frequently detected all over the world [16,17]. In the Pearl River Delta, a region with an intense manufacturing industry, PCB contents ranged from 0.30 to 202.00 ng/g with low-chlorinated PCBs as the predominant homologues [18]. In the agricultural soil of vegetable field topsoil, PCB contents reached up to 1321.3 ± 132.1 ng/g [11]. Even in remote areas of Siberia and Tibet, the detections of PCBs have also been frequently reported [19,20]. For HCBD, its level was 8.47 ng/g dry-weight in the Yangtze River Delta, higher than in the Beijing–Tianjin–Hebei region and the Pearl River Delta [21,22]. Once entering soils, these pollutants can be adsorbed by the minerals and organic matter which is affected by soil properties [23,24]. For example, coexistence of humic acid and metal cations increased PCBs sorption on the biochars in soil because of humic acid adsorption and cation complexation [24]. Meanwhile, a variety of bacteria can tolerate or degrade PCBs and HCBD in the soil environment, which could change their ecological risks [25]. Besides, soil PCBs can be accumulated in crops, even at low concentrations, presenting exposure risks to humans. Due to their high bioaccumulation, semi-volatile properties, and high toxicity, PCBs were suspected to be carcinogenic in animals and humans by the International Agency for Research on Cancer and HCBD was listed in Annex A of the Stockholm Convention [14,26].

Most studies on soil PCBs and HCBD pollution focused mainly on their pollution in open-field soil, while knowledge on their pollution status in greenhouse soil is very limited. Information on PCBs and HCBD pollution in greenhouse cultivation and comparable open-field projects have not been reported in China. In view of the popularization of greenhouse cultivation in China, the disorderly emissions of PCBs and HCBD, and the physical isolation on the soil–air exchange of greenhouses, greenhouse soils are likely to experience more serious pollution and ecological risks. The purpose of this study is to identify the contamination characteristics and sources, analyze the correlation between pollutants and environmental factors, and evaluate the ecological risks of these pollutants in greenhouse and nearby open-field soils across China. This would provide a comprehensive assessment on these pollutants when guiding greenhouse agricultural activities.

## 2. Materials and Methods

### 2.1. Sample Collection

In total, 102 topsoil samples (51 pairs, 0–20 cm) were collected from the greenhouses and nearby open vegetable fields in 20 provinces, municipalities, and autonomous regions across China (from November 2014 to February 2015). The 20 provinces, municipalities, and autonomous regions with two or three sampling sites included, Heilongjiang (HLJ), Jilin (JL), Liaoning (LN), Beijing (BJ), Hebei (HB), Shanxi (SX), Inner Mongolia (IM), Jiangsu (JS), Shandong (SD), Zhejiang (ZJ), Shanghai (SH), Hunan (HN), Jiangxi (JX), Guangdong (GD), Qinghai (QH), Gansu (GS), Xinjiang (XJ), Yunnan (YN), Sichuan (SC), and Tibet (TB). The detailed information of these sampling sites, including GPS location, crop cultivation, cultivation age, main fertilizer and soil type, was listed in Table S1. For each sampling site, soil was collected from five cores using a bamboo scoop and then was composited to form a single sample. All the soil samples were packed with aluminum foil, sealed in Kraft bags, and immediately delivered on ice to the laboratory. The remaining soil samples were stored at −20 °C for the analysis of soil properties and target contaminants [7].

### 2.2. Sample Pretreatment

Previously published methods were adopted for the extraction and cleanup procedures of PCBs [27]. In brief, all freeze-dried samples were ground and sieved through a stainless steel 75-mesh sieve. A portion of soils (5 g) was spiked with recovery surrogate PCB-65, followed by ultrasonic extraction for 30 min using hexane/dichloromethane (1:1 *v*/*v*, 40 mL). After three extractions, the extract was concentrated to 1 mL, and then subjected to cleanup using a multilayer silica column as follows (from bottom to top): 1 g silica, 4 g basic silica (1.2%, *w*/*w*), 1 g silica, 8 g acidic silica (30%, *w*/*w*), 2 g silica, and 4 g anhydrous sodium sulfate. Before use, the column was pre-washed with hexane. The extract was eluted with 100 mL hexane, and then was concentrated via rotary evaporation to a final volume of 200 μL prior to instrumental analysis.

For the extraction of HCBD, a portion of soil samples (10 g) was spiked with surrogate standard 2,4,5,6-tetrachlori-*m*-xylene, followed by ultrasonic extraction for 30 min using a mixed solvent of dichloromethane/hexane (1:1, *v*/*v*, 30 mL). The procedure was repeated two times, and all the extract was collected. Then the extract was concentrated to 1 mL via rotary evaporation and was cleaned up using a multilayer silica-Florisil composite column as follows (from bottom to top): 6 g Florisil, 4 g activated silica gel, and 5 g anhydrous sodium sulfate. Similarly, the column was pre-washed with 50 mL hexane. Finally, the extract was eluted with 150 mL petroleum ether and was thereafter concentrated to 200 μL prior to instrumental analysis [28].

### 2.3. Chemical Analysis

In all soil samples, the target PCBs included 12 dioxin-like PCBs (DL-PCBs) (including PCB-77, 81, 105, 114, 118, 123, 126, 156, 157, 167, 169, and 189), and six indicator PCBs (including PCB-28, 52, 101, 138, 153, and 180) (Table S2). The quantitative analysis of PCBs and HCBD was performed on gas chromatography/mass spectrometry (GC/MS) (7890B/5977A, Agilent Technologies, Santa Clara, CA, USA) with an electron ionization source. A DB-5 MS capillary column was used (30 m × 0.25 mm i.d. with 0.25 mm film thickness). All data were obtained in the selective ion monitoring mode. High-purity helium was used as carrier gas with a flow rate of 1.0 mL/min. Oven temperature program for PCBs was as follows: initial column temperature at 80 °C for 3 min, ramped to 150 °C at 15 °C/min and held for 2 min, increased to 270 °C at 2.5 °C/min and held for 3 min, increased to 300 °C at 15 °C/min and held for 5 min. Another program for HCBD was as follows: initial oven temperature at 80 °C for 2 min and increased to 140 °C at a rate of 10 °C/min linearly, then ramped up to 280 °C at 4 °C/min and held for 5 min.

### 2.4. Quality Assurance and Quality Control

Strict quality assurance and control measures were executed to ensure accurate identification and quantification. During the extraction process, a procedural blank, a spiked blank and a sample duplicate were carried out in parallel with each batch of samples. No targeted compound was found in the blanks. The recovery rates of PCBs and HCBD in the spiked samples ranged from 89.5% to 98.8% and from 55.2 to 76.4%, respectively. The variations in the concentration of PCBs and HCBD were lower than 20% (n = 3). For quantitative analysis, five-point standard calibration curves were employed, and the recovery rates of surrogate standard ranged from 82.5 to 99.2%. The limit of detection (LOD) of PCBs was defined on a signal-to-noise ratio of 3:1 using the lowest concentration standard which ranged from 0.03 to 0.10 ng/g. The analytes in samples were nondetectable when the results were less than the LODs.

### 2.5. Environmental Factors and Pollution Sources Analysis

To determine the correlation between environmental factors and the contamination levels of PCBs and HCBD, relevant data on these soil samples were collected from a previous report. This included the soil physicochemical properties and the levels of other co-existing pollutants [7]. The soil physicochemical properties included soil moisture (SM), soil total carbon (STC), soil total phosphor (STP), soil total nitrogen (STN), and pH value. The co-existing pollutants included organochlorine pesticides (OCPs), PAHs and heavy metals (cadmium (Cd), copper (Cu), zinc (Zn), lead (Pb)). Spearman correlation analysis was carried out to evaluate the correlations of these factors, and principal component analysis on soil PCBs compositions was performed on normalized congener data and identify their emission sources. Factors with eigenvalues greater than 1.0 were extracted and 3 principal components (>80% variance) were acquired.

### 2.6. Health Risk Assessment

The exposure noncancer and carcinogenic risks of PCBs and HCBD to human health were evaluated using the methods recommended by the U.S. Environmental Protection Agency. Generally, there are three types of exposure pathways, including soil ingestion, dermal contact, and inhalation. The average daily doses (*ADD*, mg/kg/day) via soil ingestion, inhalation and dermal contact exposure routes are estimated as follows:

(1) $$ADD_{ingest}=\frac{C_{soil}\times IRS\times EF\times ED}{BW\times AF}\times CF\;$$

(2) $$ADD_{inhale}=\frac{C_{soil}\times IhR\times EF\times ED}{PEF\times BW\times AT}\;$$

(3) $$ADD_{dermal}=\frac{C_{soil}\times SA\times AF\times ABS\times EF\times ED}{BW\times AT}\times CF\;$$

where *ADD* (mg/kg/day) is the average daily dose via non-dietary pathways, including soil ingestion (*ADD_ingest_*), inhalation (*ADD_inhale_*), and dermal contact (*ADD_dermal_*). *C_soil_* is the pollutant level in the soil (mg/kg); *IRS* is the soil ingestion rate (mg/day); *EF* is the exposure frequency (days/yr); *ED* is the exposure duration (years); *BW* is the body weight (kg); *AT* is the average lifetime exposure (days); *IhR* is the inhalation rate (m^3^/day); *PEF* is the particulate emission factor (m^3^/kg); *SA* is the dermal surface area (cm^2^/day); *AF* is the soil adherence factor (mg/cm^2^); *ABS* is the fraction absorbed dermally from the soil (unitless); *CF* is the conversion factor (kg/mg).

Hazard quotient (*HQ*) is used to estimate the noncancer risks of contaminants in agricultural soils via multiple routes, deriving from *ADD* and the specific reference dose (*RfD*) with the following equations (USEPA, 1989):

(4) $$HQ=\frac{ADD}{RfD}\;$$

where *RfD* (mg/kg/day) is defined as the daily maximum permissible level of a pollutant that will not pose noncancer risks to residents during a lifetime, including the reference dose for ingestion of contaminated food (*RfD*, mg/kg/day), *RfD_ABS_* (*RfD_ABS_* = *RfD_o_* × *ABS_GI_*; mg/kg/day) for dermal contact and reference dose of inhalation (*RfCi*; mg/m^3^) for inhalation. *ABS_GI_* is the fraction absorbed in gastrointestinal tract in the critical toxicity study (unitless).

The noncancer risks of pollutants via non-dietary and dietary pathways are presented as the hazard index (*HI*), which is calculated as follows:

(5) $$HI=\Sigma HQ_{i}\;$$

The cancer risk assessment of each pollutant is calculated as follows.

(6) RISK=ADD×SF 

where *SF* (mg/kg/day) consists of oral slope factors (*SFO*; (mg/kg/day)^−1^) for ingestion, *SFO* × *ABS_GI_* ((mg/kg/day)^−1^) for dermal contact and inhalation unit risk (IUR, (mg/m^3^)^−1^) for inhalation. Then the risks are directly summed from diverse exposure routes.

The relative parameters were shown in Tables S3 and S4. The noncancer risks of chemical contaminants were considered relatively high when *HI* > 1.0, while the risk may be negligible when the value of *HI* was below 1.0. The carcinogenic risk of chemical pollutants was considered to be very low when the risk value was below 10^−6^, low in the range of 10^−6^ to 10^−4^, moderate in the range of 10^−4^ to 10^−3^, high in the range of 10^−3^ to 10^−1^, and very high when the risk value was over 10^−1^.

### 2.7. Statistic Analysis

To compare the difference of PCBs and HCBD levels between in greenhouse and open-field soils, the statistic method of Fisher’s least significant difference was applied using Origin software (2021). The levels were set to 0 when the measured values were below LOD.

## 3. Results and Discussions

### 3.1. Levels and Spatial Distributions of Pollutants

**Figure 1:**
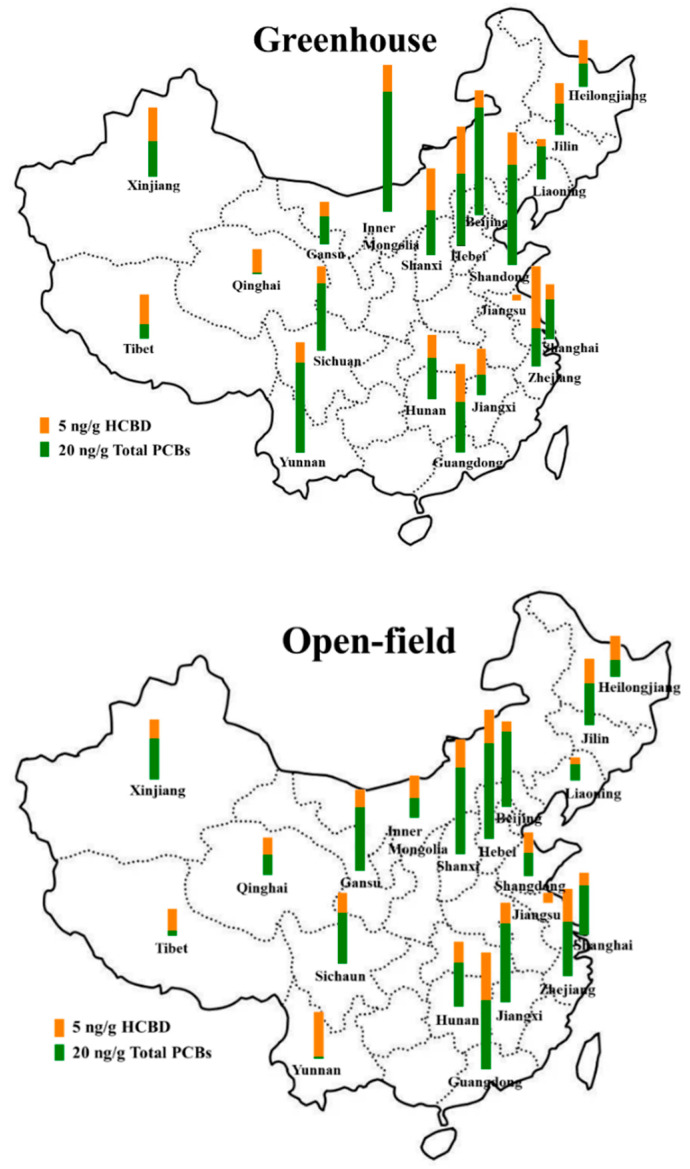
Spatial distributions of PCBs and HCBD in greenhouse and open-field soils in the investigated 20 regions of mainland China. In most regions, the concentrations of PCBs and HCBD in greenhouse soils are higher than in open-field soils.

All the contents of PCBs and HCBD were presented on a dry weight (dw) basis. At a national-scale, PCB contents ranged from <LOD to 241.22 ng/g with a mean value of 62.33 ng/g in greenhouse soils, and ranged from <LOD to 192.89 ng/g with a mean value of 51.09 ng/g in open-field soils (Table 1 and Figure 1). Furthermore, the detection frequency of PCBs was 84.31% in greenhouse soils and 76.47% in open-field soils. Despite the higher values of mean content of PCBs in greenhouse, the difference of PCBs contents was not statistically significant (*p* > 0.05) in greenhouse and open-field soil according to the analysis result of Fisher’s least significant difference (Table S5). In previous studies, PCB contents varied from 0.059 to 1.930 ng/g in paddy soils of China and varied from <LOD to 120.65 ng/g in the largest irrigation area of the Yellow River Basin, which were both lower than PCB contents in this study [29,30]. Different from their contamination levels and detection frequencies, the compositions of total PCBs (ΣPCBs) were similar in greenhouse and open-field soils. The major PCB homologues were tetra-CBs, followed by penta-CBs and hexa-CBs, which together contributed to >80% of ΣPCBs both in greenhouse and open-field soils (Figure S1). The levels of tetra-CBs reached up to 193.78 ng/g with a mean value of 27.10 ng/g in greenhouse soils and up to 110.14 ng/g with a mean value of 23.55 ng/g in open-field soils. This result was comparable to the PCB composition in the Yangtze River Delta with tetra-CBs as the major congener group in soils [31]. High-chlorinated PCBs were usually used as additives in paints and plasticizers and could enter agricultural soil by irrigation, wastewater discharge, solid-waste leakage, waste incineration, and atmospheric deposition, etc. [32,33,34]. They tend to sorb to soil in greenhouses, which would hinder the soil–air exchange and hence resulted in the higher accumulation [35,36].

For all soil samples, the HCBD contents ranged from 0.85 to 24.18 ng/g, with a mean value of 8.19 ng/g in greenhouse soils, and ranged from <LOD to 20.19 ng/g with a mean value of 6.52 ng/g in open-field soils (Table 1 and Figure 1). Compared to PCBs, the higher detection frequencies of HCBD were observed both in greenhouse (100%) and open-field soils (96.08%). Even though the mean content of HCBD levels was higher in greenhouse soils compared to open-field soils, the difference was not statistically significant (*p* > 0.05, Table 2 and Table S5). The HCBD contents in this study were comparable to that in agricultural soil of east China [21], the Yangtze River Delta [22], and southwest China [37], which both showed low contamination. In general, both PCBs and HCBD presented similar characteristics in spatial distributions in greenhouse and open-field soils which may be attributed to their pollution sources and regional environmental factors (including other pollutants and soil physicochemical properties) [38,39].

### 3.2. Regional Contamination and Source Analysis

To further identify the distribution characteristics and pollution sources in different regions, all soil sample sites were classified into seven regions: northeastern (HLJ, JL, and LN), northwestern (XJ, GS, and QH), northern (BJ, IM, SX, and HB), central (HN and JX), eastern (SD, JS, ZJ, and SH), southern (GD), and southwestern (YN, TB, and SC). As shown in Table 2, higher PCBs levels were observed in open-field soils in the northwest and south China, while higher PCBs levels were observed in greenhouse soils in other regions. The PCB levels in these regions were on the same levels as previous reports in south Jiangsu and Taiyuan [40,41]. But PCB contents in south and east China were lower than those reported by Ding et al. [42] and Zhang et al. [11], whose samples were collected from the adjacent areas near to electronic circuit board dismantling ruins, an important source of PCBs.

According to the classification of seven regions, higher PCBs levels were found in north and east China (Table 2), and thus, the contamination sources of PCBs in the two economically developed regions were analyzed. In north China, positive correlations between three pairs of PCB congener groups were presented, including tetra-CBs and hexa-CBs (r = 0.76, *p* < 0.01), penta-CBs and hexa-CBs (r = 0.76, *p* < 0.01), and penta-CBs and hepta-CBs (r = 0.76, *p* < 0.01) in open-field soils (Tables S6,S7), but no correlation in greenhouse. In east China, penta-CBs were correlated with tetra-CBs (r = 0.68, *p* < 0.01) and hepta-CBs (r = 0.57, *p* < 0.05) in greenhouse soils, and correlated with hexa-CBs (r = 0.64, *p* < 0.05) in open-field soils (Tables S8,S9). The correlations between PCB homologues indicated their similar sources/paths in greenhouse or open-field soils [43]. Also, the differences of correlations in greenhouse and open-field from two regions corresponded to the unique industrial and agricultural characteristics in different regions.

**Figure 2:**
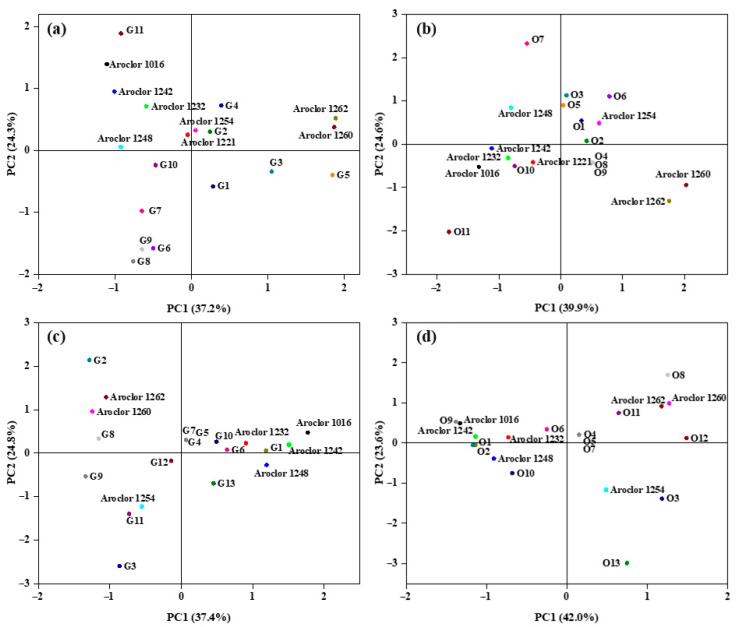
Score plot of principal component analysis of PCB homologues: (**a**) greenhouse soils in north China; (**b**) open-field soils in north China; (**c**) greenhouse soils in east China; (**d**) open-field soils in east China.

To identify their composition and the mainly emission sources, source apportionment of samples was also been performed in north and east China using principal component analysis. The PCB homologues were also compared with the Aroclor series products, including Aroclor 1016, 1221, 1232, 1242, 1248, 1254, 1260, 1262. In north China, PCB compositions were less consistent with the Aroclor series products, indicating the complexity of sources (Figure 2a,b). Both in greenhouse and open-field soils, hexa-CBs and hepta-CBs showed higher loading in PC1 and were likely to be attributed to the recycling and disposal of electrical equipment containing PCBs [44]. Tetra-CBs and tri-CBs that had higher loading in PC2 could originate from domestic coal burning, wood burning emissions, non-ferrous metal smelting and regeneration, and high-temperature incineration of industrial, as well as municipal, waste [39,40,45]. Penta-CBs with higher loading in PC3 were found mainly from oil additives and the metallurgy industry [46]. Furthermore, the similar results in both greenhouse and open-field soils suggested similar pollution sources in north China.

In east China, the compositions of PCBs were similar to the Aroclor series products in partial sample sites (Figure 2c,d). In both greenhouse and open-field soils, PCBs in the sites of G1, O1 and O2 (SH) could possibly derive from Aroclor 1242 and PCBs in the sites of G6 and O6 (ZJ) possibly derived from Aroclor 1232 [47]. Besides, PCBs composition in the sites of O9 (JX) coincided with Aroclor 1061. This indicated that PCBs in these soil samples were mainly from global contamination or from e-waste recycling containing Aroclor products. In the remaining sites, tri-CBs and tetra-CBs showed higher loading in PC1, corresponding to the main sources of domestic coal burning, wood burning emissions, high-temperature incineration of industrial and municipal waste, etc. [39,40,45]. The higher loading in PC2 and PC3 for hepta-CBs and hexa-CBs implied that they shared the same source from the recycling and disposal of electrical equipment containing PCBs [45]. In open-field soils, higher loading in PC1 for hexa-CBs and hepta-CBs, and larger loading in PC2 and PC3 for hexa-CBs and tri-CBs indicated that the recycling and disposal of electrical equipment containing PCBs was the main source [48,49]. Considering the emission sources of PCBs, these pollutants will undergo a series of migration and transformation processes and pose ecological risks. To decrease the impact, some effective measures are needed, such as establishing the process management mechanism of PCBs waste. Especially for the disposal of electrical equipment, the standard disassembly technique must be applied to prevent the emission of PCBs.

### 3.3. Correlation between Pollutants and Environmental Factors

**Figure 3:**
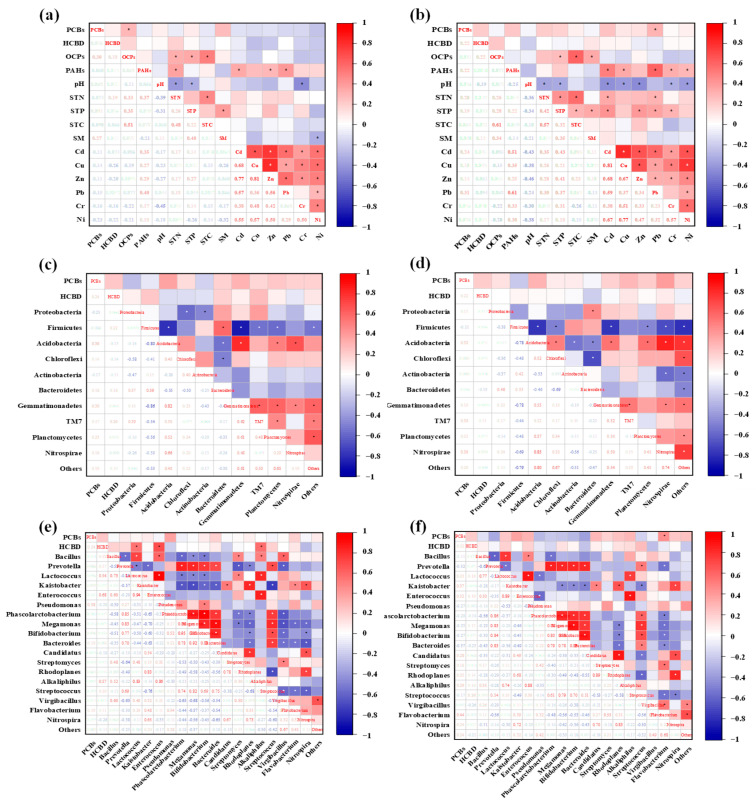
(**a**,**b**) Correlation analysis between pollutants levels and soil physicochemical properties in greenhouse and open-field soils, respectively; (**c**,**d**) correlation analysis between pollutants levels and microbial phyla in greenhouse and open-field soils, respectively; (**e**,**f**) correlation analysis between pollutants levels and microbial genera in greenhouse and open-field soils, respectively. (Note: “*” represent the significance level of *p ≤* 0.05.)

The linkages between pollutants and environmental factors were investigated through Spearman’s rank correlation analysis. At a national-scale, PCBs were positively correlated with OCPs (r = 0.30, *p* < 0.05) in greenhouse soils and Pb (r = 0.31, *p* < 0.05) in open-field soils (Figure 3a,b), indicating potential same pollution paths such as agricultural irrigation [50], industrial metal smelting, and atmospheric deposition [51,52]. Different from the positive correlation between soil organic matter and PCB contents in previous reports [53], no correlation between PCBs/HBCD and soil physicochemical properties have been found in our study; thus suggesting their diverse distribution or transformation in these regions. Meanwhile, this may also be attributed to the difference in soil particle sizes and PCB homologue groups. Compared to the smaller soil particles (75 and 100 µm), the sorption capacity of the larger particles (200 and 300 µm) for PCBs sharply decreased due to the smaller surface area of the larger soil particles [54]. The different sorption capacity of soil particles would immensely affect the partitioning of PCBs in environmental media.

PCBs compositions often present obvious change from their original states as a result of undergoing various processes of degradation, volatilization, deposition, etc. [36,55]. Under aerobic or anaerobic conditions, high-chlorinated PCBs can be degraded by aerobic microbes or dechlorinated by organohalide-respiring bacteria [56,57]. Here, the correlation analysis between pollutants levels and indigenous microbial phyla/genera have also been carried out because soil microbes play a vital role for the biodegradation of pollutants. With respect to microbial phyla levels, no correlation between ΣPCBs/HCBD and soil microbes was obtained in greenhouse and open-field soils, while only tri-CBs was positively correlated with Acidobacteria (r = 0.47, *p* < 0.05) and Nitrospirae (r = 0.74, *p* < 0.05) in open-field soil (Figure 3c,d and Figure S2a,b). With respect to microbial genera levels, hexa-CBs were negatively correlated with *Pseudomonas* (r = −0.40, *p* < 0.05) belonging to Proteobacteria, and hepta-CBs were positively correlated with *Streptomyces* (r = 0.46, *p* < 0.05) in open-field soils (Figure 3e,f and Figure S2c,d). HCBD was positively correlated with *Lactococcus* (r = 0.54, *p* < 0.05), *Enterococcus* (r = 0.68, *p* < 0.05), and *Alkaliphilus* (r = 0.57, *p* < 0.05) in greenhouse soils, respectively. However, there was no related report whether HCBD can be degraded by these microbes.

In previous studies, microbes such as Proteobacteria and Acidobacteria were reported as PCBs-degraders by performing cometabolic and direct PCB degradation [58,59]. Compared to low-chlorinated PCBs, exposure to higher-chlorinated PCBs can result in a higher abundance of Proteobacteria and Acidobacteria [25]. This was consistent with our results that these indigenous microbes were likely to degrade high-chlorinated PCBs. The abundance of Proteobacteria ranged from 3.30% to 35.60% with the mean abundance of 20.69% in greenhouse soil and ranged from 7.40% to 26.30% with the mean abundance of 17.68% in open-field soils. It was the most dominant phylum in the bacterial communities both in greenhouse and open-field soils which usually possessed aromatic compounds degrading capabilities by containing xenobiotic degradation genes [60]. Therefore, it could be a potential remediation strategy for PCBs pollution due to its high abundance and degradation capacity. *Streptomyces* could primarily contribute to plant growth and also have extreme tolerance to heavy metals such as Pb which was positively correlated with PCBs [61]. Even though few research studies have reported the degradation ability of Streptomyces for PCBs, there was no direct evidence for such degradation [62,63]. Generally, the closely relationship between soil microbes and PCBs indicated that these pollutants could pose obvious ecological impact.

### 3.4. Risk Assessments

**Figure 4:**
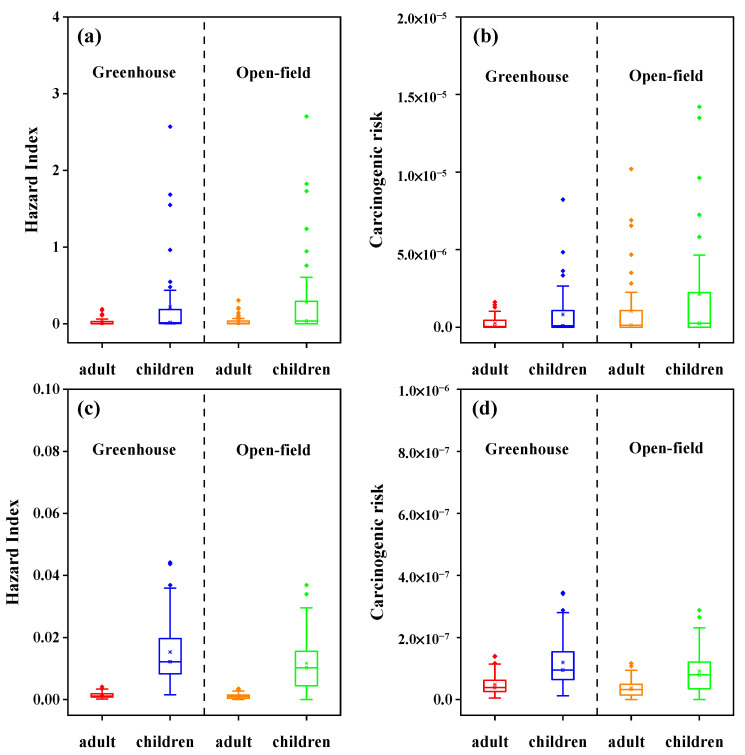
Comparison the noncancer exposure risk (**a**) and carcinogenic exposure risks (**b**) of PCBs, and the noncancer exposure risk (**c**) and carcinogenic exposure risks (**d**) of HCBD to adults and children in greenhouse and open-field soils, respectively. The health risks of ΣPCBs in open-field soils were higher than in greenhouse soils, especially for the carcinogenic risks to children (>10^−6^).

The risk assessments of pollutants were carried out from the aspects of health risk and ecological risk. The health risks of pollutants in greenhouse and open-field soils were estimated via soil ingestion, dermal contact, and inhalation exposure pathways [64,65]. As shown in Figure 4a, the noncancer exposure risks of PCBs to children and adults in open-field soils were both higher than that in greenhouse soil. Meanwhile, the noncancer exposure risks of PCBs to children were higher than to adults both in greenhouse and open-field soils. The average HI values of PCBs were 0.022 for adults and 0.222 for children in greenhouse soils, and 0.030 for adults and 0.274 for children in open-field soils, respectively. In all soil samples, lower HI values (<1.0) for adults indicated the negligible noncancer risks of PCBs to adults. But in some provinces such as BJ, XJ, SD, and GD, higher HI values (>1.0) for children indicated that the potential noncancer risks of PCBs to children cannot be negligible. Among PCB congeners, the average HI values of PCB-126 were the highest both in greenhouse soils (HI = 0.010 for adults, HI = 0.121 for children) and in open-field soils (HI = 0.016 for adults, HI = 0.146 for children), followed by PCB-81 (Figure S3). This was because the highest toxicity of PCB-126 with the minimum *RfD*_o_ and the higher content in open-field soils (Table S4). In addition, children have higher values of the average daily dose compared to adults, as children are more susceptible to ingest contaminated soils through their hand-to-mouth behavior [66]. Thus, children exposed to high-toxicity PCBs, especially in open-fields, could have higher risk of adverse health effects. For carcinogenic risks, soil PCBs also presented higher risks to children (a mean value of 2.13 × 10^−6^) and adult (a mean value of 1.03 × 10^−6^) in open-field soils compare to greenhouse soils (Figure 4b). Meanwhile, the carcinogenic risks of PCBs to children were higher than to adults. The most significant carcinogenic risks were also attributed to PCB-126 due to its toxic equivalency factor and the higher content in open-field soils then in greenhouse soils (Figure S3). Despite this, the values in large number of samples (25.5% to adults and 37.3% to children) were between 10^−6^ and 10^−4^, implying their low carcinogenic risks to human beings. For HCBD, the noncancer and carcinogenic risks for adults and children were negligible both in greenhouse and open-field soils (Figure 4c,d).

Compared with previous reports, the noncancer and carcinogenic risks of PCBs were comparable to the soils from Yellow River irrigation area and Lanzhou [50,67]; although these pollutants often presented different levels. For example, soil PCBs from IM presented higher pollution levels but lower health risks in greenhouse soils due to the toxic equivalency factor difference for different PCB congeners [29,68]. Compared to open-field, higher ratios of low-chlorinated PCBs (≤4) detected in greenhouse soils and lower risks can be attributed to the faster dechlorination of high-chlorinated PCBs characterized with high and stable moisture and temperature as well as increasing microbial growth [8,69].

For the ecological risk of PCBs, the effects-range-low (ERL) was 22.7 ng/g and the effects-range-median (ERM) was 180 ng/g according to the standard [70]. In this study, PCB contents in 33.33% sampling sites were lower than ERL in greenhouse soils and PCB contents in 39.21% sampling sites were lower than ERL in open-field soils which indicated a low ecological risk. PCB contents in 62.75% sampling sites in greenhouse and 58.82% sampling sites in open-field ranged from ERL to ERM which indicated a moderate ecological risk in these sites. Both in greenhouse and open-field, PCB contents higher than ERM were only observed in three sites (BJ, IM, and SX), indicating the high ecological risk with the 75% probability of occurrence. The moderate and high ecological risk of PCBs in greenhouse and open-field soils should take into account the value that may pose a threat to human health and ecological safety.

## 4. Conclusions

In this study, 51 pairs of surface soil samples from 20 regions were collected to investigate the contamination status of PCBs and HCBD in both greenhouse and open-field soils. At a national-scale, the mean contents of PCBs and HCBD in greenhouse soils were slightly higher than in open-field soils partly but these differences were not statistically significant. Positive correlations between PCB homologues implied their similar sources/paths in greenhouse and open-field soils mainly consisted of domestic coal burning, wood burning emissions, disposal of electrical equipment, oil additives, and the metallurgy industry. PCB compositions in some sampling sites were similar with the Aroclor series mixtures. The correlation between PCBs and soil microbes (Proteobacteria and Acidobacteria) indicated that this could be a potential remediation method. Compared to greenhouse soil, PCBs in open-field soils presented higher risks due to the higher content of high-toxicity PCB congeners. This study provided valuable information to understand the contamination status, sources, influence factors and risks of PCBs and HCBD in greenhouse and open-field soils when changing the farmland farming modes, especially for greenhouse cultivation.

## Figures and Tables

**Table 1:** Concentrations of PCBs and HCBD in greenhouse and open-field soils across China (dry weight).

	Compounds	Detection (%)	Mean (ng/g)	Median (ng/g)	Max (ng/g)	Min (ng/g)
Greenhouse	PCBs	84.31	62.33	52.22	241.22	ND
	HCBD	100	8.19	6.64	24.18	0.85
Open-field	PCBs	76.47	51.09	39.99	192.89	ND
	HCBD	96.08	6.52	5.58	20.19	ND

ND: Not detected.

**Table 2:** The concentrations and distribution of PCBs and HCBD (ng/g) in divided regions.

Different Region			PCBs	HCBD
Northeast China (n = 14)	Open-field soils	Max	103.15	12.49
		Med	32.95	5.05
		Mean	30.21	5.27
	Greenhouse soils	Max	123.86	10.89
		Med	23.05	3.93
		Mean	38.51	5.26
North China (n = 22)	Open-field soils	Max	192.90	15.68
		Med	77.35	5.84
		Mean	82.16	6.75
	Greenhouse soils	Max	241.21	23.91
		Med	97.54	8.24
		Mean	111.05	10.39
East China (n = 26)	Open-field soils	Max	143.80	20.19
		Med	43.50	4.25
		Mean	48.59	5.37
	Greenhouse soils	Max	171.21	20.19
		Med	34.75	6.48
		Mean	51.64	8.89
Central China (n = 4)	Open-field soils	Max	66.55	7.23
		Med	52.26	6.00
		Mean	52.26	6.00
	Greenhouse soils	Max	100.74	9.83
		Med	53.65	7.17
		Mean	53.65	7.17
South China (n = 4)	Open-field soils	Max	104.98	15.33
		Med	82.27	13.76
		Mean	82.27	13.76
	Greenhouse soils	Max	130.89	15.49
		Med	65.44	11.85
		Mean	65.44	11.85
Northwest China (n = 16)	Open-field soils	Max	107.92	11.40
		Med	57.27	5.31
		Mean	51.12	5.38
	Greenhouse soils	Max	85.52	16.87
		Med	8.29	6.51
		Mean	28.68	7.46
Southwest China (n = 16)	Open-field soils	Max	132.09	18.60
		Med	15.89	6.79
		Mean	23.87	8.28
	Greenhouse soils	Max	156.11	24.18
		Med	66.37	5.01
		Mean	74.52	6.88

Note: The “max and med” are the abbreviations of “maximum and median”.

## Data Availability

The data that support the findings of this study are available upon reasonable request from the corresponding author.

## Supplementary Materials

The following supporting information can be downloaded at: https://www.mdpi.com/article/10.3390/toxics11110941/s1, Figure S1: The mean concentrations of PCBs congeners in greenhouse and open-field soils; Figure S2: (a) and (b) Correlation analysis between PCB homologues and soil microbial phyla in greenhouse and open-field soils, respectively; (c) and (d) Correlation analysis between pollutants levels and microbial genera in greenhouse and open-field soils, respectively; Figure S3. The health risks of PCBs to adults and children: (a) Non-cancer risks of PCBs to children in open-field soils; (b) Non-cancer risks of PCBs to adult in open-field soils; (c) Non-cancer risks of PCBs to children in greenhouse soils; (d) Non-cancer risks of PCBs to adult in greenhouse soils; (e) Carcinogenic risks of PCBs to children in greenhouse soils; (f) Carcinogenic risks of PCBs to children in open-field soils; (g) Carcinogenic risks of PCBs to adult in greenhouse soils; (h) Carcinogenic risks of PCBs to adult in open-field soils; Tabel S1: Locations and crops cultivation of the sampling sites in this study; Table S2: The detected PCBs and their properties (Chemical book); Table S3: Parameters for adults and children in the exposure risk assessment; Table S4: Parameters associated with different pollutants in the exposure risk assessment; Table S5: Means comparisons of PCBs and HCBD concentrations in greenhouse and open-field soils; Table S6: Correlation coefficients among the PCB homologue group and soil properties in greenhouse soils of north China; Table S7: Correlation coefficients among the PCB homologue group and soil properties in open-field soils of north China; Table S8: Correlation coefficients among the PCB homologue group and soil properties in greenhouse soils of east China; Table S9: Correlation coefficients among the PCB homologue group and soil properties in open-field soils of east China.
