# Supplementary material for: Contamination Status, Environmental Factor and Risk Assessment of Polychlorinated Biphenyls and Hexachlorobutadiene in Greenhouse and Open-Field Agricultural Soils across China
Source: Toxics. 2023 Nov 20;11(11):941. doi: 10.3390/toxics11110941 (PMC10675547; doi:10.3390/toxics11110941)
Supplement: Supplementary file 1 [file toxics-11-00941-s001.zip › toxics-2686333-supplementary.pdf]

toxics

Supplementary Materials: Contamination Status, Environmental Factor and Risk Assessment of Polychlorinated Biphenyls and Hexachlorobutadiene in Greenhouse and Open-Field Agricultural Soils across China

Yaru Li, Fangwei Hou, Rongguang Shi, Xiaohua Li, Jing Lan and Zongshan Zhao

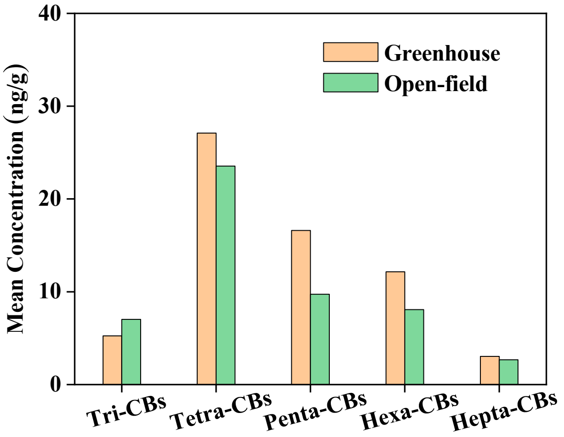

Figure S1. The mean concentrations of PCBs congeners in greenhouse and open-field soils.

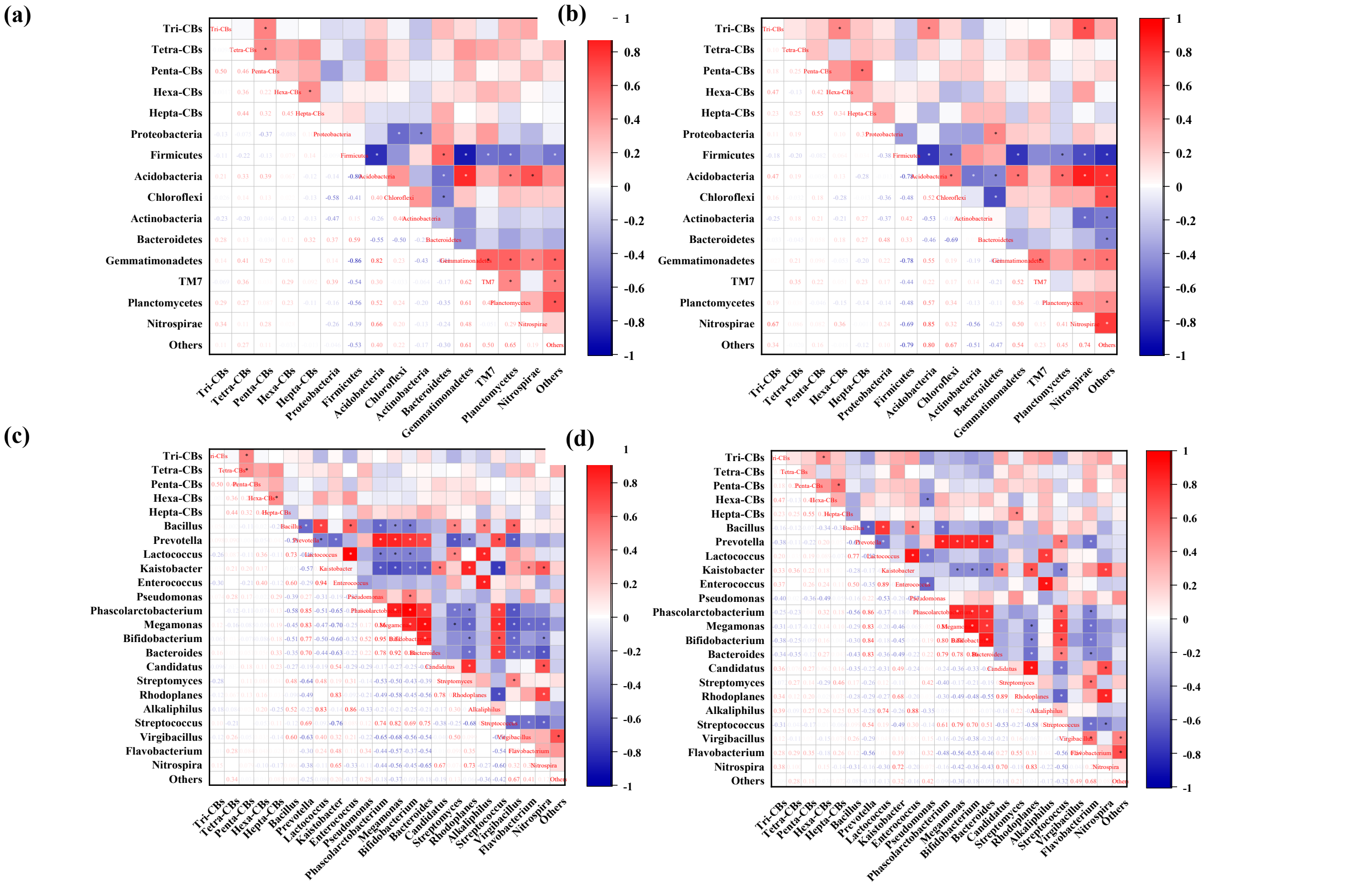

Figure S2. (a) and (b) Correlation analysis between PCB homologues and soil microbial phyla in greenhouse and open-field soils, respectively; (c) and (d) Correlation analysis between pollutants levels and microbial genera in greenhouse and open-field soils, respectively.

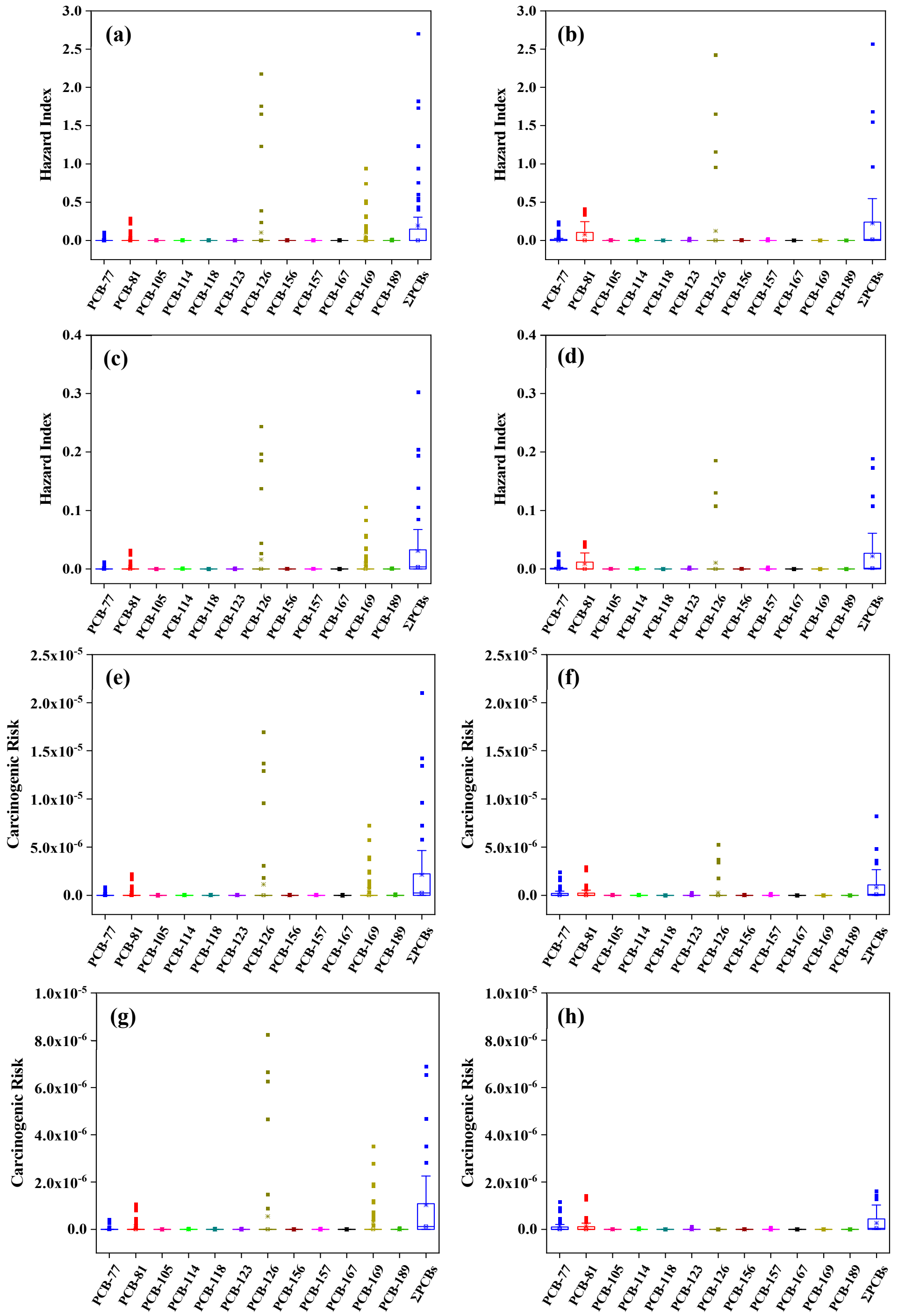

Figure S3. The health risks of PCBs to adults and children: (a) Non-cancer risks of PCBs to children in open-field soils; (b) Non-cancer risks of PCBs to adult in open-field soils; (c) Non-cancer risks of PCBs to children in greenhouse soils; (d) Non-cancer risks of PCBs to adult in greenhouse soils; (e) Carcinogenic risks of PCBs to children in greenhouse soils; (f) Carcinogenic risks of PCBs to children in open-field soils; (g) Carcinogenic risks of PCBs to adult in greenhouse soils; (h) Carcinogenic risks of PCBs to adult in open-field soils.

Table S1. Locations and crops cultivation of the sampling sites in this study.

Region	Province/Municipality/ Autonomous region	City	GPS location	Crop cultivation	Cultivation age	Main fertilizer	Soil type
Northeastern China	Heilongjiang (HLJ)	Harbin	N 45°46'19" E 126°47'37"	cucumber	2	organic	black

	Jilin (JL)	Changchun	N 45°38'7" E 126°47'1"	cucumber	2	organic	black
			N 43°44'7" E 125°22'20"	cabbage	2	chemical	black
			N 43°45'28" E 125°20'58"	cabbage	2	chemical	black
			N 43°45'28" E 125°20'58"	cabbage	2	chemical	black
	Liaoning (LN)	Dalian	N 39°13'8" E 121°43'44"	bean	3	chemical	brown
			N 39°14'1" E 121°43'28"	bean	3	chemical	brown
Northern China	Beijing (BJ)	Beijing	N 40°02'25" E 116°41'48"	leek	3	organic	brown
			N 39°44'2" E 116°22'40"	leek	3	organic	brown
			N 39°57'40" E 116°19'33"	leek	3	organic	brown
	Shanxi (SX)	Taiyuan	N 37°44'32" E 112°31'49"	cabbage	2	chemical	brown
			N 39°56'36" E 116°15'34"	cabbage	2	chemical	brown
			N 37°41'59" E 112°32'30"	cabbage	2	chemical	brown
	Hebei (HB)	Shijiazhuang	N 37°57'6" E 114°37'45"	capsaicin	2	mixed	brown
			N 37°57'25" E 114°38'7"	capsaicin	2	mixed	brown
	Inner Mongolia (IM)	Hohhot	N 40°43'20" E 111°47'13"	capsaicin	3	organic	brown
			N 40°42'48" E 111°47'56"	capsaicin	3	organic	brown
			N 40°42'10" E 111°48'54"	capsaicin	3	organic	brown
Eastern China	Shandong (SD)	Shouguang	N 36°54'26" E 118°40'29"	bean	3	mixed	brown
			N 36°52'46" E 118°51'15"	bean	3	mixed	brown
			N 36°48'47" E 118°49'35"	bean	2	mixed	brown
	Jiangsu (JS)	Suzhou	N 31°26'14" E 121°3'47"	cabbage	3	chemical	brown
			N 31°16'16" E 120°53'19"	cabbage	3	chemical	brown
	Zhejiang (ZJ)	Zhoushan	N 29°58'18" E 122°15'23"	watermelon	2	chemical	brown
			N 29°59'16" E 122°16'14"	watermelon	2	chemical	brown
			N 30°03'8" E 122°10'10"	watermelon	2	chemical	brown
	Shanghai (SH)	Shanghai	N 31°18'14" E 121°14'26"	cabbage	3	chemical	brown
			N 31°16'3"	cabbage	3	chemical	brown

			E 121°15'8"				
			N 31°12'23" E 121°12'43"	cabbage	2	chemical	brown
Central China	Hunan (HN)	Xiangtan	N 27°51'59" E 112°49'45"	cabbage	2	chemical	brown
			N 27°51'56" E 112°49'37"	cabbage	2	chemical	brown
	Jiangxi (JX)	Nanchang	N 28°44'51" E 115°54'55"	bean	2	mixed	brown
			N 28°45'71" E 115°56'09"	bean	2	mixed	brown
Southern China	Guangdong (GD)	Guangzhou	N 23°9'30" E113°22'26"	cabbage	3	mixed	red
			N 23°12'35" E 113°22'24"	cabbage	3	mixed	red
Northwestern China	Gansu (GS)	Lanzhou	N 36°08'10" E 103°22'36"	bean	3	chemical	brown
			N 35°29'19" E 103°51'38"	bean	3	chemical	brown
	Qinghai (QH)	Xining	N 36°44'14" E 101°44'43"	potato	3	organic	brown
			N 36°59'11" E 101°39'41"	potato	3	organic	brown
			N 36°58'36" E 101°39'54"	potato	2	organic	brown
	Xinjiang (XJ)	Urumqi	N 87°45'46" E 43°47'80"	tomato	2	organic	brown
			N 87°41'78" E 43°48'20"	tomato	2	organic	brown
			N 87°38'39" E 43°48'20"	tomato	2	organic	brown
Southwestern China	Yunnan (YN)	Kunming	N 24°54'43" E 102°44'52"	cabbage	2	chemical	red
			N 24°47'40" E 102°46'27"	cabbage	2	chemical	red
	Sichuan (SC)	Chengdu	N 30°34'19" E 104°09'25"	cabbage	2	mixed	brown
			N 30°32'35" E 104°00'22"	cabbage	2	mixed	brown
			N 30°33' E 104°08'26"	cabbage	2	mixed	brown
	Tibet (TB)	Nyingchi	N 29°40'16" E 94°19'58"	tomato	2	organic	brown
			N 29°37'6" E 94°23'42"	tomato	2	organic	brown
			N 29°42'32" E 94°20'6"	tomato	2	organic	brown

Table S2. The detected PCBs and their properties (Chemical book).

Detected PCB	Molecular weight	Water solubility (25 ℃)
PCB-28	257.54	116 μg/L

PCB-52	291.99	109 μg/L
PCB-77	291.99	0.549 μg/L
PCB-81	291.99	-
PCB-101	326.43	11.0 μg/L
PCB-105	326.43	-
PCB-114	326.43	15.98 μg/L
PCB-118	326.43	13.44 μg/L
PCB-123	326.43	-
PCB-126	326.43	-
PCB-138	360.88	7.29 μg/L
PCB-153	360.88	0.863 μg/L
PCB-156	360.88	5.33 μg/L
PCB-157	360.88	-
PCB-167	360.88	-
PCB-169	360.88	-
PCB-180	395.32	3.85 μg/L (20 ℃)
PCB-189	395.32	-

Table S3. Parameters for adults and children in the exposure risk assessment.

Parameter	Population	Value	Reference
IRS	Adults	100	(USEPA, 2015)
	Children	200	
EF	Adults	350	(USEPA, 2015)
	Children	350	
ED	Adults	26	(USEPA, 2015)
	Children	6	
BW	Adults	80	(USEPA, 2015)
	Children	15	
AT	Adults	9490 (non-cancer)	(USEPA, 2015)
		25550 (cancer)	
	Children	2190 (non-cancer)	
		25550 (cancer)	
IhR	Adults	13.25[a]	(USEPA, 1997)
	Children	12[b]	
PEF	Adults	1.4×10^9	(USEPA, 2015)
	Children	1.4×10^9	
SA	Adults	6032	(USEPA, 2015)
	Children	2373	
AF	Adults	0.07	(USEPA, 2015)
	Children	0.2	

[a] The mean for male and female adults; [b] The mean for different ages of children.

Table S4. Parameters associated with different pollutants in the exposure risk assessment.

Compound	*ABS*[a]	*SFO*[a]	*IUR*[a]	*RfDo*[a]	*RfCi*[a]	*ABS*$_{GI}$[a]
PCB-189	0.14	3.9	1.1	0.000023	0.0013	1
PCB-169	0.14	3900	1100	0.000000023	0.0000013	1
PCB-167	0.14	3.9.	1.1	0.000023	0.0013	1

PCB-157	0.14	3.9	1.1	0.000023	0.0013	1
PCB-156	0.14	3.9	1.1	0.000023	0.0013	1
PCB-126	0.14	13000	3800	0.000000007	0.0000004	1
PCB-123	0.14	3.9	1.1	0.000023	0.0013	1
PCB-118	0.14	3.9	1.1	0.000023	0.0013	1
PCB-114	0.14	3.9	1.1	0.000023	0.0013	1
PCB-105	0.14	3.9	1.1	0.000023	0.0013	1
PCB-77	0.14	13	3.8	0.000007	0.0004	1
PCB-81	0.14	39	110	0.0000023	0.00013	1
HCBD	—	0.078	0.022	0.001	—	1

[a] The parameters are recommend by the USEPA (2015).

.

Table S5. Means comparisons of PCBs and HCBD concentrations in greenhouse and open-field soils.

	MeanDiff	SEM	t value	Prob	Alpha	Sig	LCL	UCL
PCBs-G vs PCBs-O	-13.10635	11.0880	-1.1820	0.2400	0.0500	0	-35.1046	8.8919
HCBD-G vs HCBD-O	-1.9370	1.0564	-1.8336	0.0697	0.0500	0	-4.0329	0.1588

Note: G represent greenhouse; O represent open-field; Sig equal 1 indicates that the difference of the means is significant at the 0.05 level; Sig equal 0 indicates that the difference of the means is not significant at the 0.05 level.

Table S6. Correlation coefficients among the PCB homologue group and soil properties in greenhouse soils of north China.

	Tri-CBs	Tetra-CBs	Penta-CBs	Hexa-CBs	Hepta-CBs	∑PCBs	pH	STN	STP	STC	SM
Tri-CBs	1.000	-0.151	0.303	0.225	0.303	0.093	0.208	-0.468	-0.035	**-0.676***	0.156
Tetra-CBs		1.000	0.028	0.351	0.129	**0.907****	-0.164	-0.314	0.251	-0.091	0.114
Penta-CBs			1.000	0.037	-0.087	0.174	0.367	**-0.661***	0.138	-0.303	0.294
Hexa-CBs				1.000	0.439	**0.609***	0.209	-0.491	-0.482	**-0.727***	-0.164
Hepta-CBs					1.000	0.210	0.029	-0.114	-0.582	-0.182	-0.477
∑PCBs						1.000	0.055	-0.527	0.064	-0.391	0.118
pH							1.000	-0.382	-0.209	-0.209	0.245
STN								1.000	-0.118	**0.655***	-0.127
STP									1.000	0.309	**0.718***
STC										1.000	0.100
SM											1.000

Table S7. Correlation coefficients among the PCB homologue group and soil properties in open-field soils of north China.

	Tri-CBs	Tetra-CBs	Penta-CBs	Hexa-CBs	Hepta-CBs	∑PCBs	pH	STN	STP	STC	SM
Tri-CBs	1.000	0.137	-0.030	-0.049	0.061	0.363	**-0.645***	-0.200	0.553	-0.124	0.400
Tetra-CBs		1.000	0.307	**0.760****	0.394	**0.830****	-0.154	0.140	0.572	-0.005	-0.107
Penta-CBs			1.000	**0.761****	**0.631***	**0.688***	0.282	0.238	0.124	-0.296	-0.477
Hexa-CBs				1.000	0.438	**0.881****	0.040	0.428	0.298	0.014	-0.521
Hepta-CBs					1.000	0.516	0.151	-0.017	0.295	-0.225	-0.341
∑PCBs						1.000	-0.160	0.182	0.601	-0.146	-0.219
pH							1.000	-0.451	-0.446	-0.551	-0.155
STN								1.000	0.082	**0.755****	-0.500
STP									1.000	0.209	0.109
STC										1.000	-0.427
SM											1.000

Table S8. Correlation coefficients among the PCB homologue group and soil properties in greenhouse soils of east China.

	Tri-CBs	Tetra-CBs	Penta-CBs	Hexa-CBs	Hepta-CBs	∑PCBs	pH	STN	STP	STC	SM
Tri-CBs	1.000	0.487	0.469	0.221	0.323	0.474	-0.192	0.140	0.105	0.140	0.395
Tetra-CBs		1.000	**0.684****	0.430	0.374	**0.921****	0.049	0.211	0.043	-0.358	**0.564***
Penta-CBs			1.000	0.520	**0.569***	**0.860****	0.298	0.106	0.231	-0.538	**0.688****
Hexa-CBs				1.000	-0.022	0.482	-0.069	-0.069	0.379	-0.251	**0.680***
Hepta-CBs					1.000	**0.601***	0.305	-0.030	0.000	**-0.627***	0.192
∑PCBs						1.000	0.193	0.111	0.155	-0.525	0.675*
pH							1.000	0.050	0.132	-0.027	-0.094
STN								1.000	0.132	0.082	0.006
STP									1.000	0.209	**0.704****
STC										1.000	-0.196
SM											1.000

Table S9. Correlation coefficients among the PCB homologue group and soil properties in open-field soils of east China.

	Tri-CBs	Tetra-CBs	Penta-CBs	Hexa-CBs	Hepta-CBs	∑PCBs	pH	STN	STP	STC	SM
Tri-CBs	1.000	0.303	0.204	0.306	0.257	0.246	-0.384	0.035	0.393	-0.044	-0.044
Tetra-CBs		1.000	0.312	0.437	0.188	**0.904****	-0.214	0.335	0.520	-0.116	**0.580***
Penta-CBs			1.000	**0.636***	0.142	**0.569***	0.226	0.215	0.345	-0.237	-0.083
Hexa-CBs				1.000	0.499	**0.671***	-0.175	0.480	**0.656***	-0.028	0.202
Hepta-CBs					1.000	0.436	-0.205	0.257	0.058	-0.159	0.239
∑PCBs						1.000	-0.133	0.398	0.525	-0.188	0.473
pH							1.000	-0.412	**-0.577***	**-0.670***	-0.213
STN								1.000	0.363	0.467	0.025
STP									1.000	0.484	0.238
STC										1.000	-0.116
SM											1.000
